# Almost 2 years into the COVID-19 pandemic: an update on parental stress, parent mental health, and the occurrence of child maltreatment

**DOI:** 10.1007/s00787-023-02147-2

**Published:** 2023-02-04

**Authors:** Claudia Calvano, Lara Engelke, Anna Katharina Holl-Etten, Babette Renneberg, Sibylle M. Winter

**Affiliations:** 1 Clinical Child and Adolescent Psychology and Psychotherapy, Freie Universität Berlin, Habelschwerdter Allee 45, 14195 Berlin, Germany; 2 Clinical Child and Adolescent Psychology, Christian-Albrechts-Universität zu Kiel, Olshausenstr. 62, 24118 Kiel, Germany; 3 Clinical Psychology and Psychotherapy, Freie Universität Berlin, Habelschwerdter Allee 45, 14195 Berlin, Germany; 4 Department of Child and Adolescent Psychiatry, Psychosomatics and Psychotherapy, Charité - Universitätsmedizin Berlin, corporate member of Freie Universität Berlin and Humboldt-Universität zu Berlin, Augustenburger Platz 1, 13353 Berlin, Germany

**Keywords:** Pandemic, Parental stress, Child maltreatment, Anxiety, Depression

## Abstract

Increased parental stress, poorer mental health, and an increase in the occurrence of child maltreatment (CM) have been reported in earlier phases of the COVID-19 pandemic. However, data from later phases of the pandemic are not yet available. We conducted a cross-sectional, representative survey among 1087 parents (48.8% female; mean age 41.72 years, SD = 9.15) in Germany in December 2021. Data were compared to a previous representative sample, assessed in August 2020 (*N* = 1024), and to normative scores of the outcome measures. Predictors for the occurrence of CM were analyzed by logistic regression. Pandemic-related stress and general stress were higher and physical and mental health were poorer in the December 2021 sample than in the August 2020 sample. Occurrence rates of CM varied between 5 and 56%. Verbal emotional abuse (*n* = 607, 56%), witnessing domestic violence (*n* = 446, 41%), and emotional neglect (*n* = 435, 40%) were most frequently reported. For these subtypes, parental risk for alcohol abuse (OR 2.1–2.7) and parental recent experience of violence (OR 2.1–5.1) were the strongest predictors. Across all subtypes of CM, parents reporting child maltreatment showed poorer scores on all stress outcomes, with medium–large-effect sizes. Results confirm a high burden within the families, almost 2 years into the pandemic. Occurrence rates of a broad spectrum of CM subtypes raise further concerns for the well-being of children. Family-oriented intervention efforts are needed to stabilize families and provide targeted support. Longitudinal studies are needed for a description of families at risk for poorer outcomes.

**Supplementary Information:**

The online version contains supplementary material available at 10.1007/s00787-023-02147-2.

## Introduction

Since the beginning of 2020, the COVID-19 pandemic has been ongoing, with diverse restrictions and the re-occurrence of partial lockdowns, e.g., school closures and restrictions in outside activities. Especially, families have been facing many challenges and unforeseeable changes in their everyday life over the course of the last 2 years. A series of studies compared parents with adults without children in the household and showed higher levels of stress [1, 2] and feelings of burnout [1] among parents. Extending this evidence, a large representative study conducted in Canada in May 2020 [3] showed that caregivers (*n* = 618) compared to adults without children in the household (*n* = 2382) reported poorer mental health, e.g., suicidal thoughts and depressive feelings, and higher fear of domestic violence. Parents in their study also reported increased domestic conflict and parent–child conflicts, and in 3–19% of cases, alcohol and drug use increased [3]. Another large-scale study among 1300 mothers showed clinically increased anxiety and depressive symptoms in 35% and 32% of the sample [4]. Increased levels of parental stress during the pandemic seemed to be specifically associated with homeschooling and other restrictions, as reported in a study conducted in April–June 2020 in seven European countries among 6720 parents [5]. A representative study among 1024 parents in Germany confirmed the relation between stress in relation to lockdown measures, parental stress, and parental mental health [6].

However, not only pandemic-related restrictions and lockdown measures were associated with parental stress and parental mental health, as some studies identified specific risk groups for higher stress. A systematic review including 17 studies [7] showed that younger parents and parents who were concerned over potential job loss showed increased parental stress and poorer mental health. A recently published study among 198 parents (54% fathers) adds to this evidence, pointing to the role economic pressure plays for the link between pandemic-related tress and parental mental health [8]. Furthermore, an increase in child maltreatment, i.e., physical, emotional, or sexual abuse toward the child, or physical or emotional neglect of the child, has been widely discussed in the media and scientific literature since the beginning of the pandemic. Considering the detrimental effects of child maltreatment for child’s physical and mental health, both immediately in early ages [9] and across the lifespan [10, 11], a potential increase of child maltreatment during the pandemic adds to this significant public health topic. First studies among community samples of parents (*N* < 400) in the U.S. reported increased child abuse potential and punitive parenting [12, 13]. The above-mentioned representative study among 1024 parents in Germany extended these results: subgroups reported an increase in verbal emotional abuse (i.e., yelling at the child) and child witnessing domestic violence (i.e., observing severe parental conflict) compared to times pre-pandemic; these subgroups showed increased pandemic-related stress, parental stress, and poorer mental health [6]. Other studies confirmed that parental stress [15, 16], parental mental health [17], maternal worry, and anger about child’s behavior [14] are correlates for the occurrence of child maltreatment during the COVID-19 pandemic.

Some studies explored groups at risk for an increase of child maltreatment during the pandemic. An early study by [16] among 432 parents in the U.S. conducted in April and May 2020 showed that parental stress was related to punitive parenting and that increased alcohol consumption can fuel this relation. A cross-sectional study among 267 parents conducted in the US between June 2020 and January 2021 showed that parent’s own adverse childhood experiences (ACEs) increased the association between pandemic-related stress and negative parenting, defined as hostility, physical, and lax control [18]. The role of parental history of ACEs for an increase in child maltreatment was confirmed in our own study with a representative German sample [6]. In addition, parent’s experience of violence in adulthood, younger parent, and younger child age were also related to an increase of child maltreatment. Specifically related to the pandemic, this subgroup also reported job loss and financial loss more frequently [6].

First evidence suggests that level of burden may have fluctuated across different phases of the pandemic [19–22]. Presumably, it varies according to both the levels of lockdown measures applied at a certain time and to the cumulative burden of living in a pandemic state since the beginning of 2020. For example, in Germany, in March 2020, the first nationwide lockdown was applied, followed by lowering of restrictions up to September 2020 and a second lockdown from December 2020 till the end of February 2021. This was followed by continuously adapting measures and locally installed lockdowns during the year 2021, depending on varying criteria, for instance the local incidence rate, or individual’s vaccination status. In consequence, from the beginning of March 2021 on, the German population has still been facing certain measures, which no longer were nationwide and complete, but more chronically, partial and varying over time. The above-mentioned evidence on parental burden mainly refers to data collected between April 2020, after the first wave of the pandemic, and December/January 2020/21, when the second and third waves emerged. However, up to now, data that mirror parental burden at later times of the pandemic are still scarce.

To fill this research gap, this study aims to present cross-sectional data from a representative sample of parents in Germany collected in December 2021. The first aim is to describe the level of pandemic-related stress, parental stress, and parental subjective physical and the mental health problems experienced by parents in December 2021 and to explore demographic correlates of these outcomes. The second aim is to compare these data with (a) published reference data for the outcome measures and (b) with data from a previous representative sample of parents, assessed after the first wave of the pandemic in Germany in August 2020 [6]. Since we know of the adverse effects of long-term stressors on mental health, we hypothesized to observe higher pandemic-related stress, an increased level of parental stress, and poorer subjective physical and mental health, compared to the data collected in the August 2020 sample. The third aim is to describe the occurrence of child maltreatment as reported by the parents in the current sample, assessed in December 2021, followed by a comparison of subgroups with versus without maltreatment will be compared on the outcome measures pandemic-related stress, parental stress, general stress, and mental health. Building on these descriptive analyses, the fourth and last aim is to identify specific characteristics of the subgroup of parents reporting an occurrence of child maltreatment by logistic regression analyses: demographic variables (parent age and gender, number of children, age of the youngest child, and socioeconomic status), pandemic-specific variables (COVID-19 infections in the family, job loss, reduced working hours, and financial loss) and parent-related risk factors (current risk for alcohol abuse, self-reported mental disorder, history of childhood physical or sexual abuse, or experience of physical or sexual violence in the previous 2 months) were included as predictors for the occurrence of child maltreatment. Building on previous data [6], we hypothesized that parent and child age, job loss, and financial loss as well as a history of child abuse and the experience of violence in the previous 2 months will be significantly be related to the occurrence of child maltreatment.

## Methods

### Design

A representative survey using computer-assisted web interviews (CAWI) was conducted by INFO Marktforschungsinstitut among 1087 parents in Germany between December 10th and December 13th, 2021, using the survey software keyingress (Ingress GmbH). Participants were recruited from an active online access panel, in which they registered before for participation in research. Participants received incentives from the panel provider to reimburse them according to a set scheme. Inclusion criteria were being a parent or primary caregiver of at least one child living in the same household. Recruitment was stratified according to the German micro-census for federal state, parent gender, parent age, number of children in the household, educational level, and income. During recruitment, quota in each category was observed and filled up according to the German micro-census for the above-mentioned criteria. Thus, this recruitment strategy aimed to best represent the population of parents in Germany with respect to sociodemographic characteristics. In addition, a post-stratification weighting was applied to address disproportionalities between our recruited sample and the micro-census rate on sociodemographic factors. Through an iterative weighting procedure, the recruited sample was adjusted for parental age, parental gender, household size, parental education level, and federal state to align with the current micro-census rate. Each case was then given an individual weighting factor (rounded mean weight 1.092, rounded minimum weight 0.212, and rounded maximum weight 2.642).

### Measures

#### Sociodemographic and socioeconomic data

We collected data on parent age, gender, nationality, marital status, number of children, age, and sex of children. Data on parental educational degree, working situation, and monthly income were used to calculate the Winkler index as measure for socioeconomic status [23]. Furthermore, we included items on parental history of physical or sexual violence during childhood and adolescence, the experience of physical or sexual violence in the previous 2 months and the presence of parental mental disorder and chronic physical condition, all using an answering format yes/no. Alcohol abuse was assessed and defined by the specific module of the patient health questionnaire (PHQ), referring to the previous 2 months [24, 25].

### Pandemic-related stress

We used the Pandemic Stress Scale [26], to assess the level of burden due to several pandemic-related restrictions with 14 items (e.g., school closures, childcare closures, and social distancing). Items referred to the current burden due to the restrictions and were answered on a 5-point scale with the anchors 1 = not at all and 5 = extremely, with one additional “not applicable” option. For descriptive analyses, all answers with a 4 or a 5 were defined as “burdening”. The scale was successfully applied in a previous study [6]. Exploratory factor analysis on a large online sample assessed in Germany in August 2020 (*N* = 5020 parents) revealed 4 factors: Burden due to pandemic-related measures (7 items, e.g., school closures, home office, Cronbach’s *α* = 0.87), burden due to restrictions in social contacts (2 items, restrictions on outside activities and social distancing, Cronbach’s *α* = 0.81), burden due to restrictions on accessing treatment and services (3 items, medical treatment, psychotherapeutic treatment, and child welfare services, Cronbach’s *α* = 0.83), and burden due to health concerns (2 items, concerns about one’s own health and the health of others, Cronbach’s *α* = 0.85).

### Parental stress

We used the Parental Stress Scale [27], which includes 9 positive items on parenting (e.g., “I am happy with my parental role”), and 9 negative items (e.g., “The main reason for stress in my life are my children.”). Items referred to the past 2 months and were answered on a 5-point scale, where higher scores indicate higher parental stress. A recent evaluation of the psychometric properties of the German version in a subsample (parents with children < 16 years, *n* = 386) of a representative study sample (*N* = 2519) reported good internal consistencies (McDonalds *ω* ≥ 0.87) [28]. In this sample, internal consistency was good (Cronbach’s *α* = 0.88).

### Parental mental health

The Patient Health Questionnaire-4 (PHQ-4) [29] was used to screen for parental mental health. The PHQ-4 provides scale scores for anxiety (2 items, e.g., “Feeling nervous, anxious or on edge” or “Not being able to stop or control worrying”) and depression (2 items; “Little interest or pleasure in doing things” or “Feeling down, depressed, or hopeless”) and a total sum score. On a 4-point scale, parents rated how often they experienced symptoms, and higher scores indicate a higher symptom burden. In the validation study among a nationally representative survey (*N* = 5030) in Germany, acceptable internal consistencies for PHQ-2 (*α* = 0.78), GAD-2 (*α* = 0.75), and the total scale score PHQ-4 (*α* = 0.82) were reported [29]. Internal consistency in this sample was good (total score Cronbach’s *α* = 0.89, depression Cronbach’s *α* = 0.83, and anxiety Cronbach’s *α* = 0.84).

### General stress

We used the general stress module of the Patient Health Questionnaire [25]. The 10 items refer to potential stressful circumstances in life (e.g., relationship problems, financial concerns, and stress at work) and participants are asked to rate the degree of impairment due to the above-mentioned problems on a 3-point scale (not at all, a little, very much). According to the manual, a sum score for general stress experience is calculated (Cronbach’s *α* in this sample = 0.81).

### Subjective physical health

Using the validated 1-item measure by Benyamini et al. [30], subjective physical health was assessed (“If you were to rate your general state of health on a scale from 0 to 10 (“0” meaning “couldn’t be worse” and “10” meaning “couldn’t be better”), how would you rate your current state of health?”). In general, single-item measures yield good reliability, reproducibility, and validity [31]. This item on physical health was analyzed on a descriptive level.

### Child maltreatment and household dysfunction

Questions about child maltreatment and household dysfunction in this study were adjusted from a German questionnaire for the prospective assessment of child maltreatment in child self-report [32], which itself was derived from validated measures for adverse childhood experiences in adult retrospect [33–35]. For the purpose of our research project, we adjusted the items for parental self-report, which was successfully realized in the previous study [6]. With 11 items, we assessed the occurrence of different subtypes of child maltreatment and household dysfunction. The first item assessed the occurrence of child maltreatment on an overall level, explicitly referring to abuse, violence, or neglect as specific forms of stressful living conditions (“How often did your child experience severe stressful living conditions, e.g., violence, neglect, abuse?”). This item was followed by 8 items, assessing subtypes of child maltreatment, according to the maltreatment classification system [36], one item per subtype: verbal emotional abuse (yelling at the child more than few times), nonverbal emotional abuse (child needed to take over adult’s responsibility), physical abuse (pushing, punching, slapping or hitting the child with the fist or kicked the child with the foot), physical neglect (lack of food), supervisory neglect (paying too little attention, or not protecting the child), emotional neglect (not understanding child’s feelings nor being there for the child), witnessing domestic violence (witnessing violent fight between adults in the household), and sexual violence (touched the child at their intimate body parts or child was forced to touch another person’s body parts). In addition, we included 2 items on household dysfunction, in line with the definition on adverse childhood experiences by Felitti et al. [10] (problems related to drug and alcohol abuse in the household; problems related to adult depression or mental health problems). The items on child maltreatment and household dysfunction were successfully used in a previous study [6]. In this study, the items were introduced by “In the 2 last months, how often…” and we used a 5-point frequency scale as answering format (not at all, seldom, sometimes, often, very often). A sixth answering option was “no comment”, which constituted a low share of missing data of 0.4–2.0% across items. Besides descriptive analyses of the items, we used the answering format to create a dichotomous group variable for subgroups of parents not reporting child maltreatment (defined by answers “not at all”) and the subgroups reporting child maltreatment (defined by answers “seldom, sometime, often, or very often)”. Supplementary Material S1 includes the questionnaire for child maltreatment as used in this study.

### Data analysis

We used the weighted data for all descriptive analyses and inferential statistics. Using one-sample *t* tests, we compared the data of this sample with (a) reference scores derived from the literature (for PHQ-4, PHQ-general stress, and parental stress) and (b) with data from a representative study among 1024 parents conducted in Germany in August 2020 (PHQ-4, PHQ-general stress, and pandemic-related stress, parental stress [6]). In the two samples, measures were identical for parental mental health, parental stress, general stress, subjective physical health, and pandemic-related stress, i.e., the same questionnaires were used with the same time frame of recall. Concerning child maltreatment, the number and kind of subtypes assessed, and the item wording were also identical. However, the reference time frame and answering format differed: in the current survey, a 5-point frequency scale was used (never-very often) referring to the previous 2 months, whereas assessment in the August 2020 survey included only a yes/no answer referring to lifetime occurrence of maltreatment.

Demographic correlates of the outcome measures were explored by correlations and analyses of variance. Child maltreatment was analyzed on a descriptive level and occurrence rates in this sample were contrasted with data on lifetime occurrence, reported by the parent sample assessed in August 2020 [6]. Differences in the outcome measures (pandemic-related stress, parental stress, general stress, and parental mental health) between subgroup of parents reporting vs. not reporting child maltreatment were analyzed by ANOVAs. A stepwise logistic regression analyses were used to characterize the parents reporting an occurrence of child maltreatment (dependent variable 0 = no occurrence, 1 = occurrence of child maltreatment). Four classes of predictors were entered into the analyses: demographic predictors (parent age, parent gender, age of the youngest child, number of children in the household, and socioeconomic status), pandemic-related predictors (COVID-19 infections in the family, reduced working hours, job loss, financial loss), parent-related risk factors (parental mental disorder, parental risk for alcohol abuse, parent’s own history of childhood physical or sexual abuse, parent’s own experience of physical or sexual violence in the previous 2 months), and the stress outcomes (pandemic-related stress, general stress, parental stress, and parental mental health).

### Sample characteristics

1087 parents participated in the online survey (mean age 40.42 years, SD = 8.10, range 18–78). The youngest child in the family was on average 7.64 years old (SD = 5.24, range 0–17). Table 1 summarizes the sociodemographic data of the current sample, in comparison to the previous representative sample from August 2020 and to German micro-census data. Table 2 summarizes descriptive data related to COVID-19-related experiences, which are also compared with the data from the previous sample.

**Table 1:** Sociodemographic data and parental characteristics

	Current sample Dec 21 *N* = 1087	Sample Aug 20 *N* = 1024	Micro-census data^a^	
	*n* (%)	*n* (%)	%	
Female parent	565 (52.0%)	534 (52.1%)	–	
German nationality	1035 (95.2%)	979 (95.6%)	92.3	
Single parents	140 (12.9%)	123 (12.1%)	–	
Number of children				
1 child	584 (53.7%)	475 (46.4%)	44.7	
2 children	396 (36.4%)	422 (42.3%)	37.5	
≥ 3 children	107 (9.8%)	116 (11.4%)	17.8	
Child age groups				
0–2 years	264 (24.3%)	209 (20.4%)	15.1	
3–5 years	329 (30.3%)	247 (24·1%)	15.4	
6–12 years	635 (58.4%)	709 (69.2%)	51.7	
13–17 years	488 (44.9%)	537 (52.4%)	17.7	
Marital status				
Married or in a relationship, same household	923 (84.9%)	885 (86.4%)	87.3	
Married or in a relationship, separate households	45 (4.1%)	38 (3.8%)	–	
Not in a relationship or divorced	109 (9.9%)	94 (9.2%)	12.7	
Widowed	6 (0.5%)	7 (0.7%)	–	
School education				
Low (up to 9 years of schooling)	101 (9.3%)	83 (8.1%)	20.0	
Middle (10 years of schooling)	516 (47.5%)	365 (38.6%)	33.2	
High (up to 13 years of schooling)	452 (41.7%)	470 (55.7%)	42.6	
No school education, other, missing data	18 (1.7%)	6 (0.6%)	4.3	
Current employment status				
Not employed (e.g., retired), unemployed	98 (9.1%)	96 (9.4%)	–	
Furloughed	63 (5.8%)	51 (5.0%)	–	
In part-time employment	277 (25.4%)	276 (27.0%)	29.2	
In full-time employment	641 (59.0%)	590 (57.6%)	70.8	
In training or student	8 (0.8%)	11 (1.1%)	–	
Socioeconomic status index^b, c^				
Low	130 (12.0%)	75 (7.3%)	20.0	
Middle	670 (61.6%)	555 (54.2%)	60.0	
High	287 (26.4%)	384 (38.5%)	20.0	
Parental characteristics				
Parental risk of alcohol abuse^d^	70 (6.5%)	56 (5.5%)	–	
Parental mental disorder	161 (14.8%)	107 (10.4%)	–	
Parental history of child abuse or neglect	296 (27.2%)	238 (23.2%)	–	
Parental experience of violence in adulthood	108 (9.0%)	114 (11.2%)	–	

Data in this table are weighted data according to German micro-census (parent age, gender, number of children in the household, federal state, parental education, and household income)

^a^Population-based comparison data derived from the Micro-Census for Germany for 2019, for 2018 (school education), and for 2011 (nationality, child age groups) [37]

^b^Index calculated according to the Winkler Index [23]

^c^Population-based reference data (*n* = 12.292) for socioeconomic index derived from [23]

^d^*n* = 693 (63.8%) parents indicated that they regularly drink alcohol; of these, *n* = 70 were at risk for alcohol abuse, according to PHQ-D [25]

**Table 2:** COVID-19-related experiences

	Current sample Dec 21 *N* = 1087	Previous sample Aug 20 *N* = 1024
	*n* (%)	*n* (%)
COVID-19-related experiences		
Effects of the pandemic on health situation		
Family/household member infected with COVID-19	238 (21.9%)	22 (2.2%)
Family/household member admitted to hospital with COVID-19	60 (5.5%)	8 (0.7%)
Family/household member died with COVID-19	36 (3.3%)	4 (0.4%)
Parent belongs to risk group for severe COVID-19^a^	121 (11.1%)	103 (10.1%)
Effects of the pandemic on job situation		
Reduced working hours	315 (29.0%)	277 (27.0%)
277 (27.0%)	51 (4.7%)	55 (5.4%)
Significant financial loss	305 (28.0%)	221 (21.5%)

Data in this table are weighted data according to German micro-census (parent age, gender, number of children in the household, federal state, parental education, and household income)

^a^according to self-report

## Results

### Pandemic-related stress

Worries about the course of the pandemic (58.5%), restrictions of outside activities (54.8%), worries about others’ mental health (54.0%), and social distancing to family and friends (53.7%) were declared as burdensome by more than half of the parents. Several other areas were declared as burdensome by 40–50% of parents as well (e.g., childcare and school closures, worries about own and other’s health). A comparison of the sum score and scale mean scores between the two samples August 2020 vs December 2021 is presented in Table 3. The total score was significantly higher in the December 2021 sample than in the August 2020 sample. Concerning the subscales, a distinct pattern was observed: while the burden due to restrictions and closures was lower in the December 2021 sample, the burden due to social restrictions did not differ. The burden due to reduced accessibility of treatment and services and due to health concerns, however, was higher in the current sample, with small- to medium-effect sizes.

**Table 3:** Comparison of pandemic-related stress

	Sample Dec 21 *N* = 1087 *M* (SD)	Sample Aug 20 *N* = 1024 *M* (SD)	test statistics	*d*
Pandemic stress−sum score	35.36 (12.87)	31.97 (10.96)	*t*(1086) = 8.70	0.26*
Restrictions and closures	2.95 (1.02)	3.14 (1.00)	*t*(1083) = 6.20	0.19*
Social restrictions	3.50 (1.16)	3.45 (1.09)	*t*(1070) = 1.44	0.04^ns^
Accessibility of treatment and services	2.96 (1.15)	2.32 (1.17)	*t*(1051) = 17.99	0.56*
Health concerns	3.24 (1.19)	3.04 (1.16)	*t*(1068) = 5.63	0.17*

*p < 0.001, ^ns^ = not significant

### Parental stress

The sum score of the parental stress scale in the current sample (*M* = 37.38, SD = 10.31, range 18–82) was comparable to both reference data from the general German population (*M* = 37.1, *p* = 0.183) and with data derived from the previous study in August 2020 (*M* = 36.9, *p* = 0.074). However, parental stress in the current sample was lower than reference data from parents with children suffering from behavioral problems (*M* = 43.2, *p* < 0.001, *d* = 0.56) and lower than reference data from parent with a mental disorder (*M* = 41.9, *p* < 0.001, *d* = 0.44).

### General stress

While this sample did not express an increased level of general stress (*M* = 5.86, SD = 4.24) according to the PHQ-manual [25], the scores were significantly higher than in the sample collected in August 2020 (*M* = 5.28, *p* < 0.001, *d* = 0.14).

### Subjective physical health

The mean score for parents’ rating of their physical health was significantly lower in the current sample (*M* = 6.51, SD = 2.61, range 1–10) than in the previous sample (*M* = 6.80, SD = 2.21; *t*(1086) = 4.37, *p* < 0.001, *d* = 0.13).

### Parent mental health

**Fig. 1:**
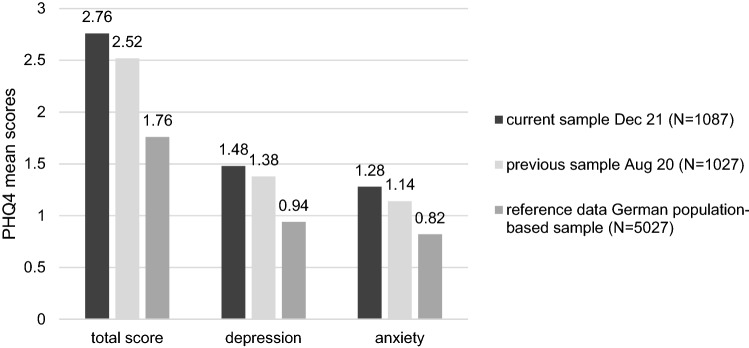
Comparison of the PHQ-4 scores for depression and anxiety with a previous representative sample of parents, assessed in August 2020 and derived from [6], and compared to a German population-based sample [25]

This sample reported significantly higher levels of anxiety and depression compared to German normative data (total score *d* = 0.35, depression *d* = 0.35, anxiety *d* = 0.30; all *p* < 0.001; [25]). The same pattern emerged in comparison to the data collected from our previous sample in August 20; however, the effect sizes were very small (total score *d* = 0.08, *p* = 0.003; depression *d* = 0.06, *p* = 0.019; anxiety *d* = 0.09, *p* = 0.001). Applying the cut-off scores for the PHQ-4 at the 95th percentile, which defines increased levels of anxiety and depression, 17.5% (*n* = 190) of the parents in the current sample scored above the 95th percentile for anxiety, 18.6% (*n* = 202) for depression and 16.2% (*n* = 176) for the total score. Figure 1 summarizes the mean scale scores for the PHQ 4 from this samples and the reference samples.

### Correlations between the outcome measures

All outcome measures (pandemic-related stress, parental stress, general stress, and mental health) were positively correlated, with highest coefficients for the correlations between stress outcomes (general stress, pandemic-related stress, and parental stress) and between general stress and mental health measures. Table 4 summarizes the results of correlation analyses.

**Table 4:** Correlations between the outcome measures in the December 2021 sample (*N* = 1087)

		Parental stress	General stress	PHQ4 total score
Pandemic-related stress		0.256	0.423	0.319
Parental stress			0.415	0.362
General stress				0.641

All *p* < 0.01

### Correlations of the stress outcome measures

To get a broader picture of our data, we explored sociodemographic correlates of the stress outcomes. Results showed small negative correlations between parent age and all outcomes (pandemic-related stress *r* = − 0.103, *p* < 0.001; general stress *r* = − 0.109, *p* < 0.001, mental health *r* = − 0.094, *p* = 0.002, and parental stress *r* = − 0.109, *p* < 0.001). There was a small negative correlation between age of the youngest child and parental stress (*r* = − 0.072, *p* = 0.017). The number of children in the household was positively correlated with pandemic-related stress (*r* = 0.112, *p* < 0.001) and parental stress (*r* = 0.079, *p* = 0.009). Mothers showed significantly poorer scores on all outcomes, with small-effect sizes (*d* = 0.07–0.28). There was no effect for SES or single parenting (data not shown).

As 22% of the sample reported COVID-19 infections in the family, we explored whether these families were more affected. Parents with a history of COVID-19 infections in the family showed higher pandemic-related stress (*F*(1, 1084) = 6.12, *p* = 0.013, *d* = 0.18), general stress (*F*(1, 1084) = 15.99, *p* < 0.001, *d* = 0.22), and poorer mental health (PHQ total score *F*(1, 1084) = 14.45, *p* < 0.001, *d* = 0.28), compared to those without, with small-effect sizes. The groups did not differ on parental stress (*p* = 0.150).

### Child maltreatment

**Fig. 2:**
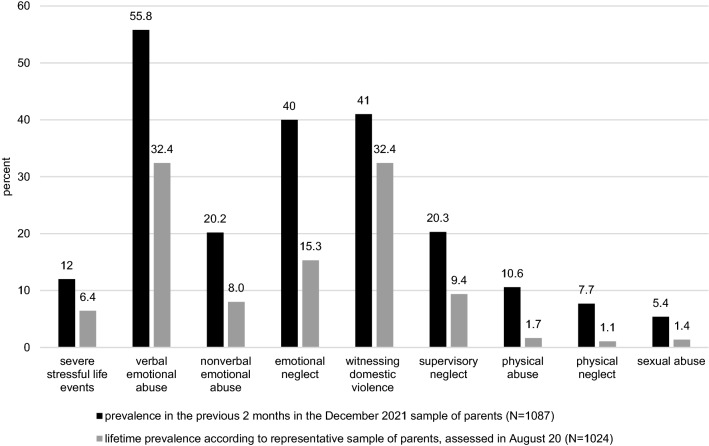
Occurrence of child maltreatments with respect to the previous 2 months, i.e., October and November 2021 in Germany as reported in the current sample (*N* = 1087) and compared to lifetime reports by a previous sample, assessed in August 2020 (*N* = 1024; [6])

Referring to the preceding 2 months, the occurrence of severe living conditions like abuse, violence, and neglect was reported by *n* = 130 (12.0%) of the parents. Among these, the occurrence was stated in 41.2% as seldom, in 34.5% as sometimes, and in 24.2% as often or very often. Concerning the eight subtypes of child maltreatment, the most frequently occurring ones were verbal emotional abuse (*n* = 607, 55.8%), witnessing domestic violence (*n* = 446, 41.0%), and emotional neglect (*n* = 435, 40.0%). Figure 2 summarizes the prevalence of child maltreatment with respect to the previous 2 months of data collection, i.e., October and November 2021 in Germany. As reference data, we added the lifetime prevalence rates reported by the August 2020 sample [6].

**Fig. 3:**
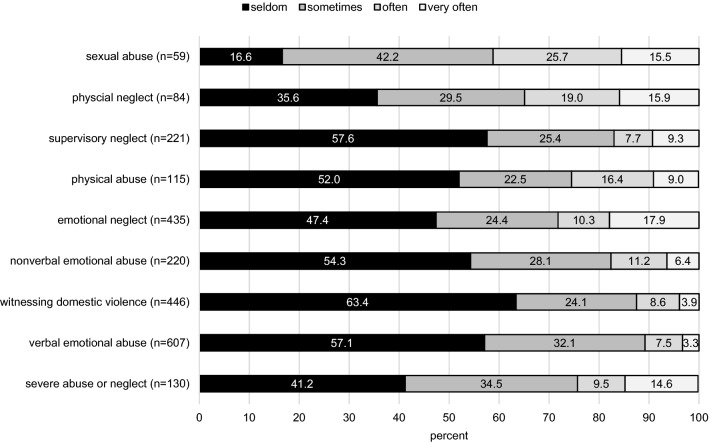
Frequency of occurrence of child maltreatment during the previous 2 months of data assessments in the current Dec 2021 sample *n* = 1087)

For the subgroups of parents reporting an occurrence of child maltreatment in the current December 21 sample (i.e., reporting an occurrence of at least “seldom”), Fig. 3 presents the frequencies in detail. While the pattern varies across subtypes, as subsample sizes do, the majority of occurrence across subtypes was stated as seldom or sometimes (58.8–89.3%). In turn, an occurrence declared as often or very often was reported in 12.5–41.2% of cases.

### Household dysfunction

Concerning aspects of household dysfunction, *n* = 81 (7.5%) parents reported the occurrence of problems related to parental drug or alcohol abuse (of these 28.7% seldom, 28.1% sometimes, 32.8% often, 10.3% very often). *N* = 187 (17.2%) reported to occurrence of problems related to an adult’s depression or mental disorder in the household (of these 42.3% seldom, 32.4% sometimes, 16.6% often, 8.6% very often). Comparison data for lifetime occurrence derived from the August 2020 sample were lower (problems related to drug or alcohol abuse 3.7%; problems related to adult’s depression of mental disorder 13.9% [6]).

We analyzed differences in pandemic-related stress, parental stress, general stress, and mental health between the groups reporting an occurrence of child maltreatment and household dysfunction (answer categories seldom-very often) vs. the group not reporting child maltreatment or household dysfunction (answer category not at all). Table 5 summarizes the mean scale scores and the effect sizes for the mean differences. For all subtypes, parents reporting maltreatment showed higher pandemic-related stress, parental stress, general stress, and poorer mental health (all *p* < 0.01, *d* = 0.19–1.59).

**Table 5:** Differences in outcome measures for parents reporting vs. not reporting subtypes of child maltreatment and household dysfunction

	Pandemic-related stress *M* (SD)	*d*	Parental stress *M* (SD)	*d*	General stress *M* (SD)	*d*	Anxiety/depression *M* (SD)	*d*
Severe maltreatment								
No (*n* = 947)	34.50 (12.59)	0.60***	36.06 (9.01)	1.18***	5.47 (3.96)	0.80***	2.56 (2.78)	0.62***
Yes (*n* = 130)	42.09 (12.70)		46.89 (10.07)		8.73 (4.83)		4.32 (3.22)	
Verbal emotional abuse								
No (*n* = 474)	34.22 (12.99)	0.19**	34.13 (9.99)	0.76***	4.86 (3.85)	0.43***	2.20 (2.74)	0.35***
Yes (*n* = 607)	36.25 (12.68)		39.86 (4.83)		6.63 (4.30)		3.20 (2.92)	
Nonverbal emotional abuse								
No (*n* = 859)	33.90 (12.57)	0.56***	35.91 (9.83)	0.70***	5.38 (3.93)	0.59***	2.51 (2.78)	0.48***
Yes (*n* = 220)	40.93 (12.34)		42.79 (10.08)		7.66 (4.66)		3.75 (3.05)	
Emotional neglect								
No (*n* = 630)	33.73 (12.44)	0.31***	34.79 (9.33)	0.63***	5.15 (3.99)	0.42***	2.46 (2.83)	0.28***
Yes (*n* = 435)	37.60 (13.05)		40.99 (10.53)		6.88 (4.31)		3.25 (2.93)	
Supervisory neglect								
No (*n* = 853)	34.54 (12.53)	0.30***	35.39 (9.42)	0.96***	5.32 (3.93)	0.62***	2.46 (2.78)	0.50***
Yes (*n* = 221)	38.41 (13.64)		44.53 (10.11)		7.84 (4.60)		3.89 (3.07)	
Witnessing domestic violence								
No (*n* = 635)	33.51 (12.86)	0.36***	34.80 (9.58)	0.62***	4.58 (3.69)	0.78***	2.11 (2.64)	0.57***
Yes (*n* = 446)	38.02 (12.31)		40.94 (10.20)		7.65 (4.24)		3.69 (2.98)	
Physical neglect								
No (*n* = 999)	60.60 (20.59)	0.58***	36.28 (9.72)	1.43***	5.57 (3.98)	0.89***	2.61 (2.81)	0.68***
Yes (*n* = 84)	72.43 (20.26)		49.99 (8.37)		9.23 (5.32)		4.55 (3.23)	
Physical abuse								
No (*n* = 895)	60.18 (20.62)	0.56***	36.12 (9.71)	1.15***	5.48 (3.97)	0.83***	2.59 (2.81)	0.57***
Yes (*n* = 115)	71.61 (19.91)		47.29 (9.53)		8.88 (4.86)		4.20 (3.07)	
Sexual abuse								
No (*n* = 1022)	60.93 (20.84)	0.53***	36.51 (9.80)	1.59***	5.69 (4.13)	0.79***	2.68 (2.87)	0.55***
Yes (*n* = 59)	71.94 (17.34)		51.90 (7.54)		8.99 (4.83)		4.25 (2.94)	
Drug or alcohol abuse in the household								
No (*n* = 1001)	60.85 (20.78)	0.41***	36.38 (9.67)	1.31***	5.56 (4.04)	0.98***	2.63 (2.84)	0.65***
Yes (*n* = 81)	69.38 (20.41)		49.15 (10.50)		9.56 (4.46)		4.49 (3.03)	
Problems related to adult mental disorder								
No (*n* = 895)	34.25 (12.70)	0.51***	35.73 (9.50)	1.06***	5.13 (3.79)	1.09***	2.23 (2.49)	1.18***
Yes (*n* = 187)	40.72 (12.32)		45.94 (10.46)		9.36 (4.33)		5.34 (3.25)	

Concerning group definition, “no” refers to the subgroup which answered “not at all”, “yes” refers to the subgroups which answered “seldom” till “very often”. Anxiety/depression refers to the PHQ-4 total score

*p < 0.05, **p < 0.01, ***p < 0.001

### Correlates of child maltreatment

Risk groups for the occurrence of child maltreatment were identified by logistic regression analyses.

The three most frequently reported subtypes of child maltreatment were verbal emotional abuse, witnessing domestic violence and emotional neglect. Results of the regression analyses are summarized in Table 6. Results for the other subtypes showed a comparable pattern (results are available upon request).

**Table 6:** Logistic regression analyses on the occurrence of verbal emotional abuse (*n* = 607), witnessing domestic violence (*n* = 446), and emotional neglect (*n* = 435)

	Verbal emotional abuse					Witnessing domestic violence					Emotional neglect				
	*B* (SE)	*p*	OR	95% CI OR		*B* (SE)	*p*	OR	95% CI		*B* (SE)	*p*	OR	95% CI	
				Lower	Upper				Lower	Upper				Lower	Upper
Demographic variables															
Parent female gender	− 0.28 (.17)	0.096	0.75	0.54	1.05	− 0.01 (0.22)	0.664	0.91	0.59	1.41	0.11 (0.18)	0.551	1.11	0.79	1.57
Parent age	0.00 (0.013)	0.983	1.00	0.95	1.03	0.02 (0.02)	0.265	1.02	0.99	1.05	− 0.01 (0.01)	0.377	0.99	0.96	1.02
Number of children	**0.25 (0.11)**	**0.022**	**1.29**	**1.04**	**1.60**	− 0.03 (0.14)	0.849	0.97	0.73	1.29	**0.27 (0.12)**	**0.018**	**1.31**	**1.05**	**1.64**
Child age^a^	− 0.03 (0.02)	0.195	0.97	0.93	1.01	0.01 (0.03)	0.626	1.01	0.96	1.07	0.04 (0.02)	0.065	1.04	1.00	1.09
SES^b^	0.10 (0.13)	0.472	1.10	0.85	1.43	− 0.08 (0.17)	0.617	0.92	0.66	1.28	− 0.09 (0.14)	0.512	0.91	0.70	1.20
Pandemic-related variables															
COVID-19 infections in family	− 0.04 (0.19)	0.838	0.96	0.66	1.40	0.20 (0.24)	0.393	1.22	0.77	1.94	**0.38 (0.19)**	**0.046**	**1.46**	**1.01**	**2.12**
Reduced working hours	**0.49 (0.18)**	**0.007**	**1.63**	**1.14**	**2.31**	0.40 (0.23)	0.078	149	0.96	2.31	0.25 (0.19)	0.181	1.29	0.89	1.85
Job loss	**0.99 (0.35)**	**0.005**	**2.70**	**1.35**	**5.40**	0.22 (0.41)	0.590	1.25	0.56	**2.77**	− 0.21 (0.38)	0.591	0.81	0.38	1.73
Financial loss	**− 0.47 (0.20)**	**0.020**	**0.62**	**0.42**	**0.93**	0.09 (0.24)	0.710	1.09	0.69	1.73	0.00 (0.20)	0.998	1.00	0.68	1.48
Parent-related risk factors															
Risk for alcohol abuse^c^	**0.73 (0.31)**	**0.018**	**2.08**	**1.13**	**3.84**	**0.80 (0.34)**	**0.019**	**2.22**	**1.14**	**4.32**	**0.98 (0.31)**	**0.001**	**2.66**	**1.46**	**4.85**
Mental disorder^d^	**0.72 (0.28)**	**0.010**	**2.05**	**1.19**	**3.53**	− 0.25 (0.34)	0.469	0.78	0.40	1.52	0.20 (0.29)	0.495	1.22	0.69	2.14
Parental history of child abuse^e^	0.09 (0.18)	0.619	1.10	0.77	1.57	0.18 (0.22)	0.411	1.20	0.78	1.87	− 0.20 (0.20)	0.307	0.82	0.56	1.20
Parental violence prev. 2 months^f^	**1.26 (0.27)**	**< 0.001**	**3.53**	**2.09**	**5.94**	**1.62 (0.29)**	**< 0.001**	**5.06**	**2.87**	**8.90**	**0.72 (0.27)**	**0.008**	**2.06**	**1.21**	**3.51**
Stress-related outcome measures															
Pandemic-related stress	− 0.01 (0.01)	0.322	0.99	0.98	1.01	0.01 (0.01)	0.583	1.01	0.99	1.02	0.01 (0.01)	0.356	1.01	0.99	1.02
General stress	0.02 (0.03)	0.422	1.02	0.97	1.07	**0.13 (0.03)**	**< 0.001**	**1.14**	**1.07**	**1.21**	0.04 (0.03)	0.174	1.04	0.99	1.09
Parental stress	**0.05 (0.01)**	**< 0.001**	**1.05**	**1.03**	**1.07**	**0.06 (0.01)**	**< 0.001**	**1.06**	**1.04**	**1.09**	**0.06 (0.01)**	**< 0.001**	**1.06**	**1.04**	**1.08**
Mental health^g^	0.04 (0.04)	0.284	1.04	0.97	1.11	0.02 (0.04)	0.600	1.02	0.94	1.11	− 0.00 (0.04)	0.929	1.00	0.937	1.07

Significant predictors are bold. Model fit for verbal emotional abuse: *χ*^2^(18) = 178.342, *p* < 0.001, Nagelkerke *R*^2^ = 0.228, Hosmer and Lemeshow *χ*^2^(8) = 14.681, *p* = 0.066. Model fit for witnessing domestic violence *χ*^2^(18) = 246.777, *p* < 0.001, Nagelkerke *R*^2^ = 0.358, Hosmer and Lemeshow *χ*^2^(8) = 5.638, *p* = 0.688; model fit for emotional neglect *χ*^2^(18) = 160.267, *p* < 0.001, Nagelkerke *R*^2^ = 0.216, Hosmer and Lemeshow *χ*^2^(8) = 8.375, *p* = 0.398

^a^Child age refers to the age of the youngest child

^b^*SES* socioeconomic status

^c^Classified according to the PHQ

^d^In parent self-report yes/no (1 = yes)

^e^Parent’s own history of physical or sexual abuse during childhood or adolescence

^f^Parent’s own experience of physical or sexual violence the previous 2 months

^g^PHQ4-total score

The only demographic variable to significantly predict CM was a higher number of children living in the household, increasing the odds for the occurrence of verbal emotional abuse (OR 1.29, 95% CI 1.04–1.60), and emotional neglect (OR 1.31, 95% CI 1.05–1.64).

Concerning pandemic-related variables, the presence of COVID-19 infections in the family was only related to emotional neglect (OR 1.46, 95% CI 1.00–2.12). Pandemic-related effects on parent’s work situation were related to verbal emotional abuse, with a distinct pattern: while reduced working hours and job loss significantly increased the odds for the occurrence of verbal emotional abuse (reduced working hours OR 1.63, 95% CI 1.14–2.39; job loss OR 2.70, 95% CI 1.35–5.40), and financial loss due to the pandemic decreased the odds (OR 0.62, 95% CI 0.42–0.93).

Two parent-related risk factors were significant correlates for all three subtypes of CM, with highest odds ratios: parental risk for alcohol abuse (verbal emotional abuse OR 2.08, 95% CI 1.13–3.84; witnessing domestic violence OR 2.22, 95% CI 1.34–4.32; emotional neglect OR 2.66, 95% CI 1.46–4.85) and parent’s own experience of physical or sexual violence during the previous 2 months (verbal emotional abuse OR 3.53, 95% CI 2.09–5.94; witnessing domestic violence OR 5.06, 2.87–8.90; emotional neglect OR 2.06, 95% CI 1.21–3.51). Parents’ self-reported mental disorder was related to the occurrence of verbal emotional abuse as well (OR 2.05, 95% CI 1.19–3.53).

Parental stress was related to all three CM subtypes, albeit with lower odd ratios (verbal emotional abuse OR 1.05, 95% CI 1.03–1.07; witnessing domestic violence OR 1.06, 95% CI 1.04–1.09; emotional neglect OR 1.06, 95% CI 1.04–1.08). Further, general stress was related to the occurrence of witnessing domestic violence with OR 1.14 (95% CI 1.07–1.21).

## Discussion

This study presents data on pandemic-related stress, parental stress, general stress, parent mental health, and the occurrence of child maltreatment, collected in December 2021 among a representative sample of parents in Germany. The aim of this paper was to present an update on these outcomes and to provide comparisons with normative scores, and with answers provided from a different representative sample of parents, assessed in August 2020, after the first 6 months of the pandemic.

First, regarding the level of pandemic-related stress in the December 21 sample, we observed a higher total score on the Pandemic Stress Scale, higher scores for burden due to restrictions in accessing medical or psychological treatment, accessing child welfare services and for burden due to health concerns, compared to answers in the August 2020 sample. The burden due to restrictions on a social level was comparable between the two samples; the burden due to other, mainly job-related restrictions and closures, was lower in the current sample. This may be explained by changes in state-wide measures. In Germany, the second nationwide lockdown ended in spring 2021, and since then, widespread closures of offices, schools, and childcare were no longer implemented. This may have resulted in a lower burden specifically in this area. However, as schools and childcare facilities have repeatedly and locally been closed due to local infections, reactions and adjustment in parent’s and family everyday life were demanded again (e.g., organization of home office, quarantine measures within the family). This may in turn contribute to the higher burden overall across diverse areas. The relevance of pandemic-related stress was further underlined by positive correlations between the sum score for the pandemic stress scale with all other outcome measures. As measures for pandemic-related stress vary across studies [3, 5, 6, 38–40], direct comparisons with other samples are limited.

We observed a moderate level of parental stress in this sample. This is in line with findings from our August 2020 sample, and with other studies conducted at the beginning of the pandemic [6, 15, 41, 42]. Concerning parental mental health, participants in December 21 reported significantly higher anxiety and depression than population-based normative scores and the previous sample; however, the effect sizes were very small. The rate of parents with increased levels of anxiety (17.5%) and depressive symptoms (18.6%) in December 21 is lower than pooled prevalence rates reported in the literature. For instance, a recent meta-analysis (including 158 studies published until December 2020) reported a pooled 25% prevalence rate for depression and 27% rate for anxiety for the general population [43]. It should be noted that across studies, measurements vary and comparisons are limited. In our study, key symptoms of anxiety and depression were assessed with the screening measure PHQ-4. Due to the cross-sectional design, our data do not imply a rise of mental health problems. While a large-scale meta-analysis points to the global rise of depression and anxiety during 2020 [44], no pooled data derived from longitudinal studies are available yet, that analyze the development of parental mental in the long-term course of the pandemic up to 2021/2022.

The current sample also reported higher general stress and poorer physical health compared to the August 2020 sample. As cut-offs are not available and measures differ, comparisons with other studies are limited. The burden reported by parents in December 2021 may mirror the burden due to the ongoing pandemic [45, 46]. Our data also showed that mothers reported more stress than fathers, which is in line with common literature [47, 48]. Further, younger parents and parents with younger children reported higher levels of stress, which also is in line with the previous findings [6]. However, our data are only cross-sectional and provide an initial update; longitudinal studies including later time points in the pandemic will provide deeper insights into the ongoing burdens in future research.

Concerning the occurrence of child maltreatment, verbal emotional abuse (56%) and witnessing domestic violence (41%) were the most frequently reported subtypes, in line with previous data on parent-reported child maltreatment during the pandemic [6]. In addition, emotional neglect showed a comparably high 40% prevalence in our study and was among the three most frequently reported subtypes. Comparison data on child maltreatment rates during the pandemic are scarce. One cross-sectional online study among 283 parents conducted in USA in spring 2020, after the first wave of the pandemic, yielded comparable results [49]. Lee et al. [49] asked parents about the occurrence of abuse and neglect in the previous 2 weeks of data collection and report a 61.8% prevalence of verbal aggression toward the child, 23% emotional neglect, 19.9% physical punishment, and 12.4% physical neglect. Our rates are partly comparable (emotional abuse, physical neglect), partly higher (emotional neglect), but also partly lower (physical abuse). The time point of assessment (earlier vs. later phases of the pandemic). Methodological differences in time frames and item wordings as well as cultural differences might account for differences and need to be considered.

In this paper, we compared the current results with the lifetime prevalence data published in Calvano et al. [6] from the August 2020 survey, where we used the same measurement for child maltreatment, with the same subtypes and same item wordings. Across all subtypes, we now observed higher prevalence rates than the lifetime reports in 2020. For instance, in the current sample, 12% of the parents indicated the occurrence of severe stressful living conditions (abuse, violence, and neglect) in the previous 2 months, compared to a 6.4% lifetime prevalence reported in the August 2020 sample. Also, regarding household dysfunction, occurrence rates were higher in the current sample. While the same measure for child maltreatment was used in both studies, i.e., same number and kind of subtypes and same wording of items, the time frames and answering format differed: in the August 2020 sample, we asked for lifetime occurrence of child maltreatment with a yes/no-answering format. In contrast, in this sample, parents were asked for the frequency of occurrence during the previous 2 months on a 5-point scale *not at all—very often*. These methodological differences are important to keep in mind when comparing the data from the two different samples in this paper.

Besides the lifetime prevalence data reported by the parent sample from our previous study [6], other population-based lifetime prevalence data on child maltreatment may also help to put our results into context. Witt et al. [50] provide lifetime prevalence rates for child maltreatment in Germany, retrospectively assessed among adults aged 14–94 years with the Childhood Trauma Questionnaire. Within their sample, the data derived from the group of adolescents and young adults aged 14–19 years show the shortest retrospective recall interval and that way, the closest approximation to the prevalence of recent maltreatment, as it is assessed in our study by the parent report for the previous 2 months. While the prevalence rates in the Witt et al.’s study [50] of sexual abuse (5.6% vs. 5.4% in our study) and physical neglect (5.6% vs. 7.7%) are comparable with our data, rates for physical abuse (7.0% vs. 10.6% in our study) and emotional neglect (6.3% vs. 40% in our study) are lower than the data from our study. Taking together, when comparing our data with other parent-reported prevalence data on the occurrence of child maltreatment during the pandemic [50], results point to a high prevalence of child maltreatment during the pandemic, with the most consistent pattern for emotional abuse. When comparing our data with general prevalence rates on child maltreatment, patterns are more inconsistent.

Irrespectively of the comparisons, rates reported in our study are high on each CM subtype. In this regard, we wish to emphasize three aspects regarding child maltreatment presented in this paper: (1) the high prevalence rates of child maltreatment across all subtypes are alarming, even if some of the items of the measure mirror a restricted range of maltreatment with comparatively lower severities; (2) a detailed analysis showed that the frequency of CM occurrence varied from seldom to very often; (3) while it is not possible to evaluate the frequency of occurrence with respect to harmfulness or severity, note that across subtypes, the majority of our sample declared the occurrence as seldom or sometimes. While sexual abuse showed the comparatively lowest rate (*n* = 59, 5.4%), this form of maltreatment showed the highest proportion describing the occurrence with often or very often—a definite reason for additional concern.

We compared the families reporting vs. not reporting child maltreatment in our study on pandemic-related stress, parental stress, general stress, and parental mental health. Across subtypes of child maltreatment, the subgroup of parents reporting child maltreatment showed poorer outcomes, which is in line with previous data from earlier phases of the pandemic [14–16]. The results also mirror the pattern in our representative study conducted in Germany in August 2020 [6], where subgroups of parents reporting both lifetime occurrence and an increase, since the beginning of the pandemic showed poorer scores on all outcome measures.

We aimed to identify families at increased risk for an occurrence of child maltreatment during the pandemic. Summarizing the results, parent-related risk factors played the major role for the occurrence of child maltreatment: parent’s recent experience of physical or sexual violence (OR 2.1–5.1) and parental risk for alcohol abuse during the previous 2 months (OR 2.1–2.7) were related to all three subtypes of child maltreatment. Specifically related to the occurrence of witnessing domestic violence was the presence of a self-reported mental disorder (yes/no-answering format; OR 2.05). Self-reported presence of a mental disorder was a stronger predictor than parental anxiety and depressive symptoms, as assessed with the PHQ-4. The latter did not significantly contribute to the prediction of CM in this study. These findings are in line with other studies conducted during the COVID-19 pandemic, which reported an association between parent’s experience of violence [6, 17], parental alcohol abuse [16, 54], the presence of parental mental disorder [17], and the occurrence of maltreatment toward the child. However, and contrary to hypotheses and to findings from other studies [6, 18], a history of own childhood physical or sexual abuse—reported by *n* = 296 parents in the current sample—was not a significant predictor for CM in this study.

Concerning pandemic-related risk factors, the strongest relations were found for the occurrence of verbal emotional abuse, albeit with a specific pattern: job loss (OR 2.70) and reduced working hours (OR 1.63) were positively correlated with the occurrence of verbal emotional abuse verbal toward the child. Financial loss during the pandemic showed an opposite direction (OR 0.62). We can only speculate about this pattern of results. One aspect might be that being at home more often, because of job loss or reduced working hours, might fuel tension and the tendency to yell at the child. Financial loss, in turn, might have an impact on other levels than child maltreatment, as this predictor did not have a significant positive correlation with any of the CM subtypes.

Concerning the stress outcomes pandemic-related stress, general stress, and parental stress, the pattern of results was less consistent: among this group of predictors, parental stress was the only outcome which was related to the occurrence of all three maltreatment subtypes, albeit with comparatively lower odd ratios (OR 1.05–1.06). According to the hierarchical procedure, we can conclude that when taking demographic, pandemic-related, and parent-related risk factors into account, parental stress has an additional, but weak contribution to the occurrence of child maltreatment. General stress and pandemic-related stress showed weaker and less consistent effects. While the results of the regression analyses point to certain risk groups and mainly confirm other findings in the context of the pandemic, it should be noted that our findings are only cross-sectional and remain correlational. Longitudinal data and multivariate analyses are needed to provide a deeper knowledge on risk groups and the interactions between risk factors and outcomes.

The assessment of child maltreatment we applied in this study and in the previous one [6] needs to be discussed with respect to strengths and limitations. One strength is that we assessed a variety of child maltreatment subtypes and the time frame refers to the previous 2 months. Due to this short time of recall, the data indicate recent maltreatment and may have been less biased than longer term recall. Further, the missing data rate on our maltreatment measure was very low (0.3–2.0%), pointing to both acceptance and feasibility of our approach. In the most prevalent forms of maltreatment, the item wording reflects milder forms of maltreatment, which may have contributed to the high response rate. One limitation regarding our approach might be the potential underestimation of maltreatment reported only by parents (themselves). However, other studies also successfully used parental self-report for the assessment of the occurrence of child maltreatment [9], especially during the COVID-19 pandemic [12, 51]. In general, using caregiver report is one approach for the assessment of child maltreatment, which should ideally be complemented by youth self-report and official physician or chart data [52]. However, as a recent systematic review on informant discrepancies in child maltreatment assessment [53] underlines: sources often do not agree.

The following other limitations of this study need to be considered, which refer to generalizability of the current sample and the comparability of the two different samples: recruitment for this survey was stratified according to the micro-census in Germany and the data were additionally weighted according to the micro-census. However, sample characteristics were still slightly deviated from the micro-census—mainly with respect to SES, as families with low SES were underrepresented. Further, only German-speaking parents were eligible for study participation. Thus, generalization of the current data on non-German-speaking families, families with migrant background and families with low SES is limited. In addition, the two studies which were compared in this paper differed in the recruitment mode: in the first survey in August 2020, we conducted a mixed-mode design with 40% telephone-based assessments and 60% online assessments. The second survey only used online assessments. We can only speculate about the effects of recruitment in this study, as known factors for bias like age and gender [55] were predefined by the micro-census and, accordingly, the sample was comparable on all sociodemographic variables. Besides differences in recruitment, the two samples differed in some distinct characteristics. While differences in sociodemographic variables seem minor, pronounced differences were observed in COVID-19-related experiences. As expected, a higher number of families were affected by infections in December 2021. Exploratory analyses suggest that this rather affected general stress and mental health measures, as specific pandemic-related stress and parental stress did not differ between the families affected and not affected by COVID-19 infections.

Pandemic-related burden, poor mental and physical health, high general stress, and an occurrence of child maltreatment in 5–56% of cases indicate a tremendous burden within the families. Other studies conducted during the pandemic focused on child mental health and report an increase in mental health problems in children since the beginning of the COVID-19 pandemic [56, 57]. Based on the assumption that parental burden might go along with youth psychopathology [58], the need to support parents is additionally emphasized. Parent’s pandemic-specific stress, general stress, mental health, and aspects of child maltreatment are important areas for family-oriented intervention and prevention efforts. Specifically concerning child maltreatment, prevention efforts need to take parental risk factors into account. Our data suggest that parents at risk for alcohol abuse, parent’s recent experiences of violence, presence of a mental disorder, as well as job loss and reduced working hours due to the pandemic constitute a target group for preventive and interventive efforts. Several prevention approaches have been applied during the first year of the pandemic, e.g., applying a CBT-based approach [59], or a resilience-oriented approach [60]. While evaluation data are on the way in many trials, the first results confirm the effectiveness for a universal, resilience-oriented prevention program with positive effects on family level, on parenting and on child psychological well-being as well [60]. In line with these approaches and efforts, implemented during the first year of the pandemic, the multiple burdens observed in our sample call for an awareness for the ongoing burden the parents are still facing. Further implementation, evaluation, and dissemination of family-oriented prevention programs are urgently needed for buffering the effects of the ongoing pandemic on both parents and children. Child welfare and prevention of child maltreatment need to be among our top first priorities now and post-pandemic, to limit long-term negative sequelae for children and the parents and to promote the family’s mental health.

## Supplementary Information

Below is the link to the electronic supplementary material.

Supplementary file1 (PDF 83 kb)

## Data Availability

Data are available upon request to the corresponding author.
