# Supplementary material for: Almost 2 years into the COVID-19 pandemic: an update on parental stress, parent mental health, and the occurrence of child maltreatment
Source: Eur Child Adolesc Psychiatry. 2023 Feb 4;32(12):2593–609. doi: 10.1007/s00787-023-02147-2 (PMC9899111; doi:10.1007/s00787-023-02147-2)
Supplement: Supplementary file 1 — Supplementary file1 (PDF 83 kb) [file 787_2023_2147_MOESM1_ESM.pdf]

Supplementary material S1

Article title: Almost two years into the COVID-19-pandemic: an update on parental stress, parent mental health, and the occurrence of child maltreatment

Journal: European Child & Adolescent Psychiatry

Authors: Claudia Calvano PhD[1, 2], Lara Engelke MSc[3], Anna Katharina Holl-Etten[2], Babette Renneberg PhD[3*], & Sibylle M. Winter MD[2*]

*shared senior authors

Affiliations: [1] Freie Universitat of Berlin, Department of Education and Psychology, Clinical Child and Adolescent Psychology and Psychotherapy

[2] Charité - Universitätsmedizin Berlin, Corporate Member of Freie Universität Berlin, Humboldt-Universität zu Berlin, Berlin Institute of Health (BIH), Department of Child and Adolescent Psychiatry, Psychosomatics and Psychotherapy

[3] Freie Universität of Berlin, Department of Education and Psychology, Clinical Psychology and Psychotherapy

Corresponding author: Claudia Calvano, PhD**,** Email: claudia.calvano@fu-berlin.de

Supplementary material 1: Assessment of child maltreatment

A modified and shortened version of the pediMACE (Isele, 2016) interview was applied. All items referred to actions either by the parents (supervisory neglect, emotional neglect) or by the parents or other adults in the household (verbal emotional abuse, nonverbal emotional abuse, physical abuse, physical neglect, witnessing domestic violence), except the item on sexual abuse, which referred to any adult or clearly older person.

Instruction:

The following questions relate to the topic of domestic violence.

Parenthood is not always easy, as life circumstances are stressful and children can be challenging. We also know that such situations can affect education and education not always runs perfectly. Since you have the best overview, we ask for your answers here.

It is important that you answer the questions as honestly as possible. All information in this survey is anonymous.

The following questions refer to the previous 2 months.

Maltreatment subtype	How often...	**Not at all**	**Seldom**	**Sometime**	**Often**	**Very often**
Severe stressful living conditions	... have there been severe stressful living conditions (violence, neglect, abuse) for the child/ children?					
Verbal emotional abuse	... has at least one adult who lives in the household with the child/ children ever yelled at the child/ children?					
Nonverbal emotional abuse	... did the child/ children in your household have to take over a responsibility, which should only be taken over by a significantly older child or adult?					
Emotional neglect	… have neither the father nor the mother (or other main caregivers / parental-like persons) been able to understand the feelings of the child /children or could be there for them?					
Physical abuse	… have there been situations in which at least one adult who lives in the household with the child/ children intentionally					

	pushed, pinched, slapped or hit the child/ children with the fist or kicked the child/ children with the foot?
Supervisory neglect	… have there been situations in which at least one parent paid too little attention or did not protect the child/ children?
Physical neglect	… have there been situations in which the child/children in your household did not have enough to eat?
Witnessing domestic violence	... have there been situations in which your child/ children experienced how adults who lived with the child/children argued violently?
Sexual abuse	... have there been situations in which an adult person has touched your child's/ children's intimate parts of the body without the child/ children wanting this or forced the child/ children to touch another person's body?
Household dysfunction: problems related to drug abuse	... have domestic problems occurred in connection with an alcohol or drug problem?

Household dysfunction: problems related to parental mental disorder	... have there been domestic difficulties related to depression or mental illness?
Experience of physical or sexual violence in the previous. 2 months	... have there been situations in which you yourself were intentionally hit, kicked or otherwise experienced bodily harm or were forced to perform unwanted sexual actions?

Reference:

Isele, D. R. (2016). *The role of Adverse Childhood Experiences (ACEs) in clinical disorders: A new assessment tool and evaluation of links with borderline personality symptoms* . Published Doctoral Thesis, University of Konstanz, Germany.
